# End-to-End Deep Graph Convolutional Neural Network Approach for Intentional Islanding in Power Systems Considering Load-Generation Balance

**DOI:** 10.3390/s21051650

**Published:** 2021-02-27

**Authors:** Zhonglin Sun, Yannis Spyridis, Thomas Lagkas, Achilleas Sesis, Georgios Efstathopoulos, Panagiotis Sarigiannidis

**Affiliations:** 1 0 Infinity Ltd., Imperial Offices, London E6 2JG, UK; sunlf281@gmail.com (Z.S.); yannis@0infinity.net (Y.S.); achilleas@0infinity.net (A.S.); george@0infinity.net (G.E.); 2 Department of Electrical and Computer Engineering, University of Western Macedonia, 50131 Kozani, Greece; psarigiannidis@uowm.gr; 3 Department of Computer Science, International Hellenic University, 65404 Kavala, Greece

**Keywords:** intentional islanding, deep learning, graph convolutional networks, graph partition, spectral clustering, power system, load-generation balance

## Abstract

Intentional islanding is a corrective procedure that aims to protect the stability of the power system during an emergency, by dividing the grid into several partitions and isolating the elements that would cause cascading failures. This paper proposes a deep learning method to solve the problem of intentional islanding in an end-to-end manner. Two types of loss functions are examined for the graph partitioning task, and a loss function is added on the deep learning model, aiming to minimise the load-generation imbalance in the formed islands. In addition, the proposed solution incorporates a technique for merging the independent buses to their nearest neighbour in case there are isolated buses after the clusterisation, improving the final result in cases of large and complex systems. Several experiments demonstrate that the introduced deep learning method provides effective clustering results for intentional islanding, managing to keep the power imbalance low and creating stable islands. Finally, the proposed method is dynamic, relying on real-time system conditions to calculate the result.

## 1. Introduction

In recent years power systems have seen a significant growth in capacity and their complexity has inevitably increased. As a result, unexpected incidents that may occur due to natural disasters or human faults, can lead to considerable oscillations in the power system, often leading to partial or total outages. Given the detrimental social and economic consequences that arise in these cases, there is an increasing need for efficient control strategies that alleviate the impact of such events and protect the system from cascading failures.

### 1.1. Intentional Islanding

When a power system is subject to major disturbances leading to unstable operation, rapid-responding mechanisms are essential for restoring the stability and prevent a blackout. Intentional islanding is a procedure that aims to partition the power grid into isolated, sustainable islands, which has been shown to stabilise the system following a critical incident [1]. The goal is to decide which transmission lines should be disconnected, in order to create stable islands that are smaller in size, and thus easier to control, enabling a fast system restoration. To guarantee the system stability, effective islanding must take into consideration the coherence of the grouped generators, while minimizing disruptions and load shedding [2].

A key enabling factor of intentional islanding in power systems is the integration of microgrids, which offer several benefits, as they can operate both connected to the main grid, or disconnected in islanded mode, operating autonomously [3]. Microgrids allow the smooth integration of distributed energy resources that can match the local demand, and offer the possibility of decentralised control, thus enhancing privacy and improving energy delivery and system stability [4]. In such power systems, major disturbances that can be caused by unexpected physical disasters or human errors, involving the loss of generators, can often lead to cascading failures and result in a blackout. Modern power infrastructures should incorporate smart mechanisms that help the system respond rapidly to such events and restore quickly into a stable state [5].

The problem of intentional islanding is generally categorised in the non-deterministic polynomial-time hardness problems. For large and complex power systems, the search space for the optimal solution is vast and most approaches for dealing with this problem are based on optimization theory [6,7]. Several existing studies also formulate the islanding problem as a mixed-integer linear programming (MILP) problem [8,9,10], distinctly handling power balance in each created island for a feasible solution. In these approaches, the decision on the island margins and generator configuration is taken with respect to the balance of the load-generation and the minimization of load shedding, while ensuring that system constraints are not violated [11].

Intentional islanding is often translated into a graph partitioning problem, by modelling the power grid using a graph representation. Typically, graph-based methods involve cutting the minimum number of lines in the graph. This approach is usually preferred, as the islanding result tends to provide exceptional connectivity performance. The power grid can be converted into a directed and weighted graph based on real-time topology and load flow, and then progressively reduced to a smaller size, which can be partitioned according to grouping information of the generators [12]. A common issue that arises in these approaches, is the large search complexity during the graph partition. This problem can be addressed by utilising the multilevel kernel k-means procedure for fast islanding [13], which consists of three stages: aggregation, partition, and retrieval. The graph is firstly aggregated into a low resolution for coarse partitioning and the multilevel kernel k-means method is used to revise the results of each retrieval step.

### 1.2. Graph Neural Networks

Machine learning (ML) is an area of artificial intelligence that utilises existing datasets to make predictions on unseen data. A machine learning model is initially trained using a known dataset, and the generalization performance of the model is then evaluated on the unseen data. Inside the broader family of machine learning lies deep learning, which is based on artificial neural networks that simulate the structure of brains. Several deep architectures have been introduced in the literature, notably after the arrival of LeNet-5 [14], one of the earliest convolutional neural networks (CNNs) that inspired the evolution of deep learning. A highly influential neural network in the field of image classification is AlexNet [15], which was presented in 2012 and introduced the ReLU and Dropout layers in the network architecture. Other notable deep neural networks include the VGG [16] that made a huge impact after AlexNet in computer vision and the ResNet which managed to gain significant accuracy from a considerably increased depth, while being easier to optimise [17].

The structures mentioned above are mainly designed for Euclidean distance data and target visual recognition applications, utilising matrices that store the pixels of an image. This type of structure is not appropriate for capturing graph data, as required for the representation of a power system. The key difference between image and graph data lies in the definition of the connection and image data can be considered a special case of graph data. For this reason, the research community has explored ways to incorporate graph data into deep architectures, an endeavor that led to the graph neural network (GNN) paradigm. Let graph G=(V,E), where *V* is the set of vertices and *E* is the set of edges. Being an adaptation of convolution neural networks, the graph neural network encodes information of individual elements as vertices, and their interrelation as edges, aiming to capture patterns within the graph.

Graph neural networks are usually designed either on a spatial or a spectral domain, with recent frameworks bridging the gap and demonstrating the equivalence of the convolution process regardless of the design domain [18]. Based on the spatial design, the architecture aims to define a receptive field from the neighbouring vertices and extract the spatial features to finish the convolution operation on the graph. The spectral design, introduced in [19], utilises a weight matrix to calculate the feature map dimension for the convolution, but in cases where the feature dimension is large, training becomes too slow and there is no locality. This issue can be addressed as demonstrated in [20], reducing the number of parameters to be trained and introducing local information into the graph neural network, by having spectral filters with linear evaluation complexity.

This paper explores the potential of using graph neural networks to address the problem of intentional islanding in real-time. The key contributions are the following:

- The proposed method is the first to incorporate an end-to-end deep learning solution for the intentional islanding problem. It incorporates four loss functions in total.
- The load-generation imbalance is minimised at each island using a deep learning loss formulation, enhancing the stability of the power system after the islanding process.
- A loss function is defined to determine the cluster for each bus in the system.
- A loss function is also defined to ensure the coherency of generators in the formed islands and avoid loss of power supply.
- An additional loss function is defined to balance the number of nodes in each partition.

The introduced solution offers the advantage of addressing the intentional islanding problem with increased time efficiency, providing a real-time solution in a few seconds. The solution is aldo dynamic and is based on the real-time status of the system; it does not rely in predetermined lines that can be disconnected. The different loss functions aim to determine robust islands to relieve frequency problems in smaller clusters, leading to a faster system restoration. A possible shortcoming of the proposed method, is that since it is based on a graph neural network, it might not always be optimal. However, due to the very short amount of time in which the solution is offered, it can prove crucial in emergency situations and in fact often preferred to an optimal solution that will require much longer to be calculated, since rapid islanding will be required in order to prevent further outages and therefore minimise the need for large black-start units.

The rest of the paper is organised as follows: Section 2 discusses existing techniques for intentional islanding in the literature. The problem formulation and the deep learning approach are thoroughly described in Section 3. Section 4 presents the evaluation of the proposed solution and analyses the results of the islanding experiments on several test cases. Finally, Section 5 concludes the paper.

## 2. Related Work

Several studies have been conducted in search for effective grid splitting strategies, targeted at avoiding cascading breakdowns during critical incidents. Most islanding solutions are proposed with respect to the objective of having minimum power disruption or achieving a load and generation balance. A common approach involves spectral clustering techniques, which are often used in power grid graph representations to group the nodes and determine the formed islands. A multi-layer graph modelling approach is introduced in [21], where spectral clustering is used to define the islands based on certain constraints for coherent grouping of the generators. A similar approach is followed in [22] by introducing a two-stage, multi-layer constrained clustering method. The proposed model utilises a number of criteria, namely active power, reactive power, and apparent power, each resulting in different islanding results accordingly. A simulation evaluation demonstrated that this technique can assist the effective operation of the grid during a critical event, while also preserving reliability and security aspects.

A spectral k-embedded clustering algorithm is introduced in [23], where an islanding solution is provided through solving the generalised eigenvalue problem. The authors determine the appropriate number of clusters by coherently grouping the generators, and they use the graph representation of the power system to obtain the weight and Laplacian matrices. Following the normalisation of the eigenvectors, those related to the k-1 lowest eigenvalues are selected and the algorithm to calculate the islanding solution is applied. A similar approach is followed in [24], proposing a constrained spectral clustering algorithm, which is able to achieve the intentional islanding solution that results in minimum power flow disruption, while establishing islands that include coherent generators. In the introduced method, the corresponding eigenproblem is solved once, irrespective of the formed island quantity, resulting in improved efficiency in large-scale power systems.

The method in [25] utilises a two-step spectral clustering algorithm which uses the minimal power flow disruption as the objective function. The authors define a requirement related to the coherency of the grouped generators, in order to avoid situations where islands with isolated load are formed. During the first phase, the normalised clustering method groups the generators and the final islanding solution is calculated in the second phase using constrained spectral clustering. The authors in [26] model the islanding problem as an MILP optimisation problem. Utilising graph theory, the splitting strategy is translated into a graph partition problem and considering the subgraph connectivity constraints, a recursive procedure is used to determine the optimal islanding solution that involves the minimum number of lines to be disconnected.

An intentional islanding algorithm based on MILP formulation is also introduced in [10], that guarantees minimum power-flow disruption for any amount of formed islands. The complexity of the MILP problem is reduced by utilising a pre-processing mechanism, that aims to determine which trees connect each coherent generator class with the minimum quantity of nodes. In terms of computational complexity the above methods achieve reasonable results, but they do not satisfy dynamic constraints in the clusterisation process, which may lead to the creation of unstable islands, thus increasing the probability that a blackout will be caused eventually. In addition, such approaches cannot achieve polynomial time and require computational times exponential in order when having as objective the minimisation of the power imbalance during the islanding process. A similar approach is proposed in [27], developing an MILP formulation to search the optimal paritioning strategy. The authors rely on the Benders Decomposition method, where the problem is divided into a relaxed MILP master problem and an LP subproblem. In addition, the number of line disconnections required is reduced by adding a switching constraint and the coherent groups of generators are selected using a set of linear equations.

Following a three-stage method, the work in [28] tackles the intentional islanding problem by utilising a self-adaptive graph simplification technique. During the first stage, an appropriate islanding cutset search area is obtained from the initial grid graph model. Then, a recursive merge algorithm is used in the obtained search area, to determine the islanding schemes that result in minimal load-generation imbalance. In the third and final stage, a depth-first searching technique is checking the outputs of the previous stage and determines the final cutset, based on the avoidance of low-voltage issues. A control strategy that allows intentional islanding operations in distributed power systems is introduced in [29], where the authors propose an intelligent load-shedding algorithm, able to maintain the voltage and current within desirable levels during the islanding mode. A method for transitioning back to grid-connected operation is also introduced, capable of avoiding a hard transient during the reconenction.

The advent of machine learning has led to novel approaches in the field of graph partitioning, potentially supporting novel solutions in the area of intentional islanding. Efficient training of large graph neural networks plays a key role towards realising effective solutions in this regard. An algorithm for training very deep networks in a short period of time and using small amounts of memory is introduced in [30], named Cluster-CGN. The algorithm exploits a graph clustering structure to associate a block of nodes with a dense subgraph and limit the search within that section. The authors demonstrate that with proper optimisation, a deeper architecture can achieve a higher accuracy and F1 score. In [31], a generalisation of GNNs is proposed, namely the k-GNN which is based on the k-dimensional Weisfeiler–Leman algorithm. Experiments demonstrate that this model is able to recognise more graph properties and outperforms traditional GNNs in extensive learning tasks.

Current intentional islanding approaches miss the powerful fitting and generalization capabilities offered by deep learning architectures. In this regard, this paper proposes a novel deep graph neural network method that addresses the intentional islanding problem in an end-to-end manner, by ustilising deep learning loss formulations.

## 3. Deep Learning Based Method for Intentional Islanding

This section analyses the proposed deep learning solution for the problem of intentional islanding in power systems. The introduced method does not require labelled data for training the model; the whole process is conducted end-to-end in an unsupervised manner. Section 3.1 describes the incorporation of deep learning in the graph partition problem and Section 3.2 introduces this solution into the islanding problem.

### 3.1. Graph Partition

Deep learning approaches have been recently applied in graph partition problems. The solution proposed in this paper is based on the Generalisable Approximate Partitioning (GAP) framework for graph partitioning [32] and the normalized min-cut problem [33]. The islanding problem can be formulated as a minimum cut problem as follows:

(1) $$cut\left(S_{k},{\hat{S}}_{k}\right)=\sum_{v_{i}\in S_{k},v_{j}\in {\hat{S}}_{j}}e\left(v_{i},v_{j}\right),$$

where $S_{k}$ is the k-th set of a given graph, $\hat{S_{k}}$ represents the the remaining sets except $S_{k}$ and $e(v_{i},v_{j})$ is the edge between vertex $v_{i}$ and $v_{j}$. When referring to multiple sets, the cut problem can be rewrited as:

(2) $$cut\left(S_{1},S_{2},S_{3}\ldots S_{g}\right)=\frac{1}{2}\sum_{i=k}^{g}cut\left(S_{k},{\hat{S}}_{k}\right)$$

The problem of minimum cut has been extensively studied in the literature, with the normalised cut representing a distinctive direction:

(3) $$Ncut\left(S_{1},S_{2}\ldots S_{g}\right)=\sum_{k=1}^{g}\frac{cut(S_{k},{\hat{S}}_{k})}{vol(S_{k},V)},$$

where $vol\left(S_{k},V\right)=\sum_{v_{i}\in S_{k},v_{i}\in V}e\left(v_{i},v_{j}\right)$ is the total degree of nodes from $S_{k}$ in the graph *g*. This formulation represents the cost of the cut divided by the total number of edges in the subset.

#### 3.1.1. GAP

The GAP method addresses the normalised cut problem using deep learning optimisation, transforming the minimum cut problem into a deep learning format as follows:

(4) $$L_{cut}=\sum_{reduce\_sum}\left(Y⊘\Gamma \right){\left(1-Y\right)}^{T}⊙A+\sum_{reduce\_sum}{\left(1^{T}Y-\frac{n}{g}\right)}^{2}$$

In the above equation, the first term is the normalized cut, *Y* is defined as an n∗g dimension matrix that represents the output of the neural network, where *n* is the number of nodes and *g* is the number of partitions. *A* is the adjacency matrix and finally Γ is calculated by *Y*:

(5) $$\Gamma =Y^{T}D,$$

where *D* is the vector that represents the degree of the nodes. The second term is the balance cut that restricts the number of nodes in every partition.

In our proposed implementation the balance cut loss is relieved into the following formulation:

(6) $$L_{balance}=max\left(0,\frac{n}{g}-\sigma 1^{T}Y\right),$$

where σ is the hyper-parameter to adjust the relief degree.

#### 3.1.2. Min-Cut

The minimum cut problem is formulated as follows:

(7) $$L=L_{c}+L_{o}=-\frac{Tr(S^{T}\hat{A}S)}{Tr(S^{T}\hat{D}S)}+\left|\right|\frac{S^{T}S}{\left|\right|S^{T}S{\left|\right|}_{F}}-\frac{I_{K}}{\sqrt{K}}\left|\right|,$$

where $\left|\right|\cdot {\left|\right|}_{F}$ is the Frobenius norm, *S* is the prediction of which cluster the node belongs to, *A* and *D* indicate the adjacency matrix and degree matrix, respectively. $I_{k}$ is the multiplication of the balanced clustering result *S* and it’s transpose matrix, where *S* predicts exactly the balanced number of nodes in every cluster N/g. Tr() represents the average operation: $Tr\left(\right)=\frac{1}{K}\sum_{k=1}^{K}$.

*L* is the total loss function for the minimum cut and $L_{c}$ is the cut loss term, which is easy to result in a local minima solution. For instance, after optimisation, $L_{c}$ tends to assign all vertices in a binary cluster result, regardless of how many classes we assign. $L_{o}$ is the orthogonality penalty term so that nodes from different clusters are orthogonal and the number of nodes in every cluster is of similar size.

### 3.2. Islanding Using Deep Learning

From the above formulations it is observed that deep learning methods have been considered for the graph partition problem; however, these approaches have not yet been introduced into the intentional islanding problem. In this paper, an end-to-end deep learning solution for intentional islanding is proposed, based on the GAP and min-cut loss functions presented in the above subsections. Furthermore, the load-generation imbalance of each island is minimised, using the following loss function:

(8) $$L_{load-gen}=\frac{1}{K}\sum_{k=1}^{K}\left|YB\right|,$$

where *Y* is the output matrix from the neural network with dimension n∗g and contains the probability of every node belonging to each partition. *B* is the *n* dimension vector that represents the load-generation result approximated from the state estimation. After the minimization of this loss function, the deep learning model can determine the partition in which each node should belong.

To further ensure the stability of the power system, the requirement that every cluster should have at least one generator is defined, avoiding situations where there is no power supply after the cut has been applied. The proposed loss function for assigning a specific generator to every cluster is formulated as follows:

(9) $$L_{gen}=softmax\_cross\_entropy\left(p,Y_{gen}\right),$$

where $Y_{gen}$ is the matrix indicating the buses that are connected to the source generators, *p* is a vector that ranges from 0 to *g*, and softmax_cross_entropy is a classical loss function for multi-class optimisation loss, which consists of two parts, softmax and cross-entropy. The Softmax operation is defined as:

(10) $$a_{i}=\frac{e^{z_{i}}}{\sum_{k}^{e^{z_{k}}}},$$

where $z_{k}$ are the elements of the output vector. The cross-entropy loss function is defined as:

(11) $$J=-\sum_{i}y_{i}\ln a_{i}$$

With the cross-entropy loss, the predicted value tends to have a similar distribution with the ground-truth value. The total loss function is given by:

(12) $$L_{total}=\alpha L_{cut}+\theta L_{balance}+\beta L_{load-gen}+\gamma L_{gen}$$

In the above equation, α, β, γ and θ are the hyper parameters to adjust the balance of the total loss. A more accurate number of nodes in one cluster can be obtained by assigning a higher weight to θ.

### 3.3. Coarse-Fine Adjustment

Due to vast complexity on large graphs, a merging technology was introduced that allows to reassign the isolated buses to their nearest cluster. After getting the prediction results from the neural network, the neighbour information for every bus is checked. If there is a neighbouring bus with the same clustering outcome, the process continues to the next bus. Otherwise, the distance of features from the adjacent buses is calculated and the bus is reassigned to the nearest cluster.

There are several approaches to find the nearest cluster. In the proposed solution, owing to the generalization performance of features from the unsupervised neural network, the nearest bus is calculated by the following formulation:

(13) $$V_{nearest,i}=argmin||F\left(v_{i}\right)-F\left(V_{j}\right)\left|\right|,$$

where $V_{nearest,i}$ is the bus selected as the nearest node of bus $V_{i}$, and $V_{j}$ is the candidate bus adjacent to $V_{i}$. F(·) denotes the feature extracted from the first linear layer of the graph neural network, and ||·|| is the euclidean distance of different nodes. Besides the above method, the nearest bus can also be selected from the physical location defined by the power system.

### 3.4. Graph Representation of the Power System

**Figure 1:**
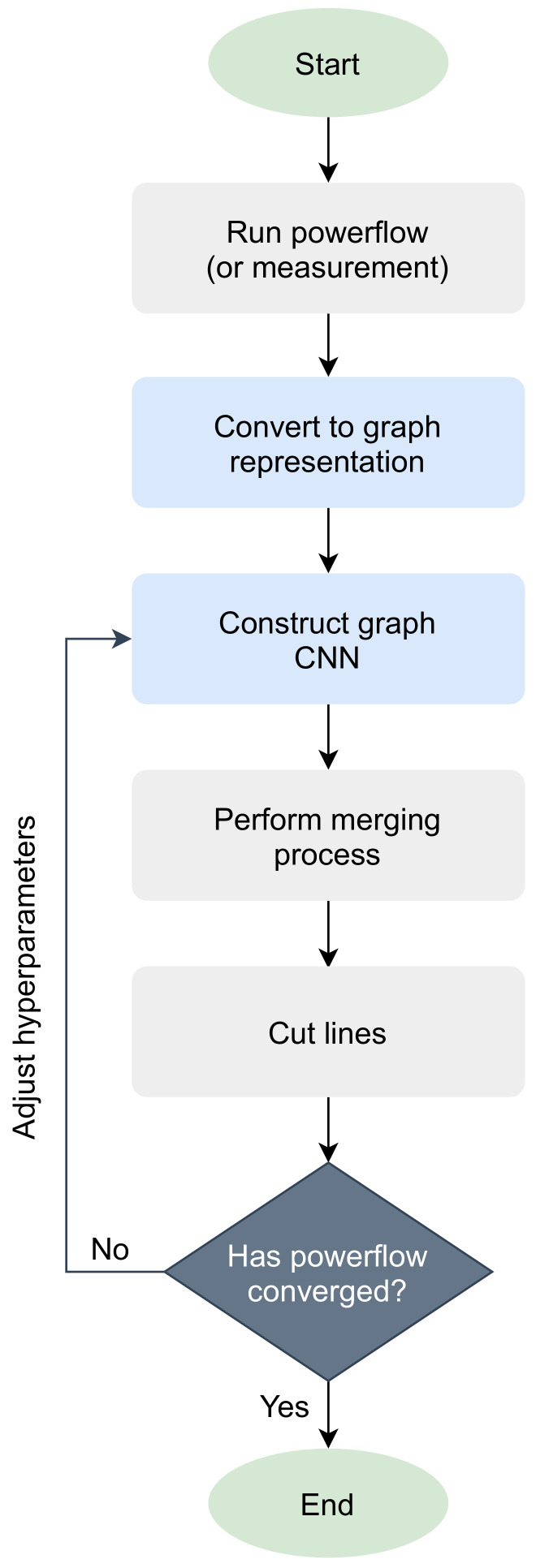
Flow chart depicting the proposed pipeline of the method for intentional islanding.

In order to use the deep learning methods for intentional islanding, the power system has to be translated into a graph representation. The pipeline of the devised graph CNN approach is presented in the flowchart of Figure 1. The main elements of focus are buses, lines (switches are also considered as lines) and generators. In the graph format, powerflow analysis is first executed on the given power grid, extracting the output, which in case of buses is voltage magnitude, voltage angle and power demand, while in case of lines it is active power flow, reactive power flow and adjacency information. For generators, the index of buses that connect to each one is required. The buses are regarded as the nodes of the graph and weighted lines represent the edges, where the weight is the active power flow or the adjacency information. The graph neural network is then built to train the model to convergence and the prediction result is fixed by merging the single isolated vertices into another vertex. Finally, the line cut is applied and powerflow analysis is conducted to check whether the network converges. In case of non-convergence, the hyper-parameters are adjusted and the model is retrained.

## 4. Evaluation Experiments

The key goal of this section is to discuss the evaluation of the proposed solution for the problem of intentional islanding. It provides a description of the model implementation, the simulation results indicating the performance of the introduced methods and an ablation study.

**Figure 2:**
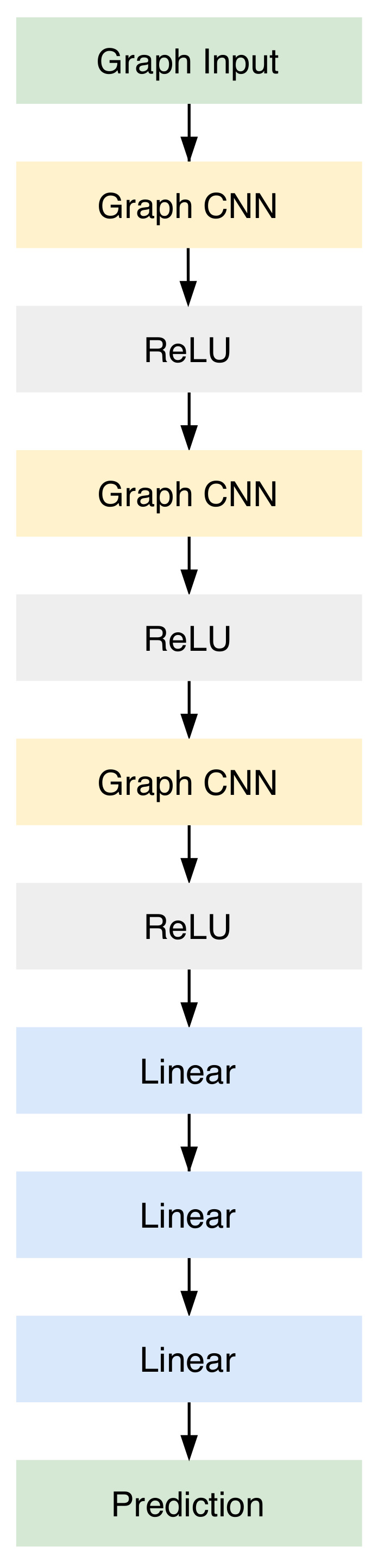
Model structure, showing the layers that comprise the proposed neural network for intentional islanding.

The first step towards addressing the intentional islanding problem is the conversion of the power system into a graph format. After conducting powerflow analysis on the power system, the extracted results are used for the graph representation. As mentioned in the previous section, several objective functions are used for optimisation in the proposed deep learning method. In order to utilize the graph representation to finish the clusterisation, a new structure was defined. As displayed in Figure 2, the model is comprised of three layers of graph CNN, along with three ReLU layers and finally, three linear layers. The purpose of ReLU layers in deep learning architectures is to overcome the vanishing gradient problem and they usually allow the model to learn more quickly. The linear layers are used in the end to scale the output to the dessired number of clusters. The output dimensions of each layer are listed in Table 1, where *g* is the number of clusters.

### 4.1. Model Implementation

**Figure 3:**
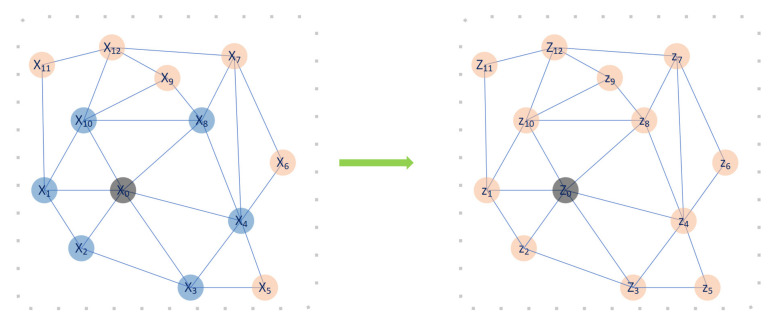
Example of a graph CNN’s function. For every node (coloured black in this instance), it takes the one-hop or two-hop neighbor as the receptive field. The blue nodes indicate the selected receptive field or neighbouring nodes, while the yellow ones are not selected for the black node. Graph CNNs use a filter to perform convolution on the selected nodes $X_{i}$, resulting in the new status $Z_{i}$, where the blue nodes are considered to calculate the black value in the right subfigure.

While traditional CNNs can only capture information on euclidean distance, graph CNNs provide convolution on graph data. By incorporating these into the architecture, the whole prediction process is end-to-end and only requires the graph representation of the power system to get the clustering result. Figure 3 demonstrates an example of how graph CNNs utilize neighbour connection information as topology representation. Graph CNNs are designed for each node which is colored black in the figure for each time and the neighbour nodes are chosen as the input feature of the Graph CNNs. The convolution on the spatial domains equals multiplication in the spectral domain, which is represented by the Laplacian matrix of the graph. Finally, the calculated result is stored in the black node. Following this procedure, all the nodes in the candidate graph are updated similarly.

For the training and testing process, Pytorch [34] was used as the deep learning platform, together with Tensorflow [35] and Mxnet [36]. The above platforms were selected since they utilize the GPU to accelerate the whole training process. In the proposed implementation, every power system was trained for 10,000 iterations which took approximately 7 min for training on a system with a GTX-2060 GPU and an i7-8750H CPU. The result was achieved in 0.1 s. In addition to the deep learning platform, the pandapower library [37] was used as the power system simulation tool, since it provides a large number of functions for power systems, including powerflow and state estimation.

### 4.2. Simulation Results

**Figure 4:**
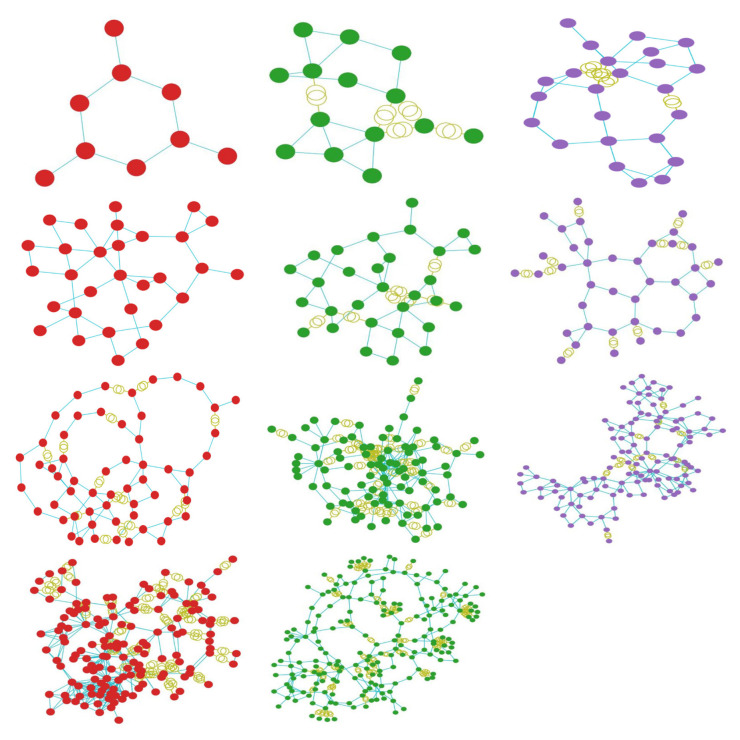
The test cases used in the simulation experiments. Starting from top left and moving horizontally, the images display the power system of cases: 9, 14, ieee 24, 30, ieee 30, 39, 57, 89, 118, 145 and 200.

To measure the islanding performance, the proposed methods were evaluated on the test samples provided by the pandapower library, which include adaptations from actual power systems. Cases 9, 14, IEEE 24, 30 (representative of an actual system in New England, USA), IEEE 30 (approximation of the American Electric Power system as it was in December 1961), 39 (from actual New England Power System), 57, 89, 118 (portion of the American Electric Power System in the Midwestern US), 145, and 200 were chosen as the simulation cases. Figure 4 shows the aggregated images of the above cases. In the conducted experiments, the number of clusters was set as a hyperparameter and was configured to an aprroximate value, which is later optimally established by the algorithm and in most cases was determined to be 3. To demonstrate the effectiveness and efficiency of the proposed methods, the imbalance of load-generation power was evaluated in every cluster, along with the number of lines to be cut.

**Figure 5:**
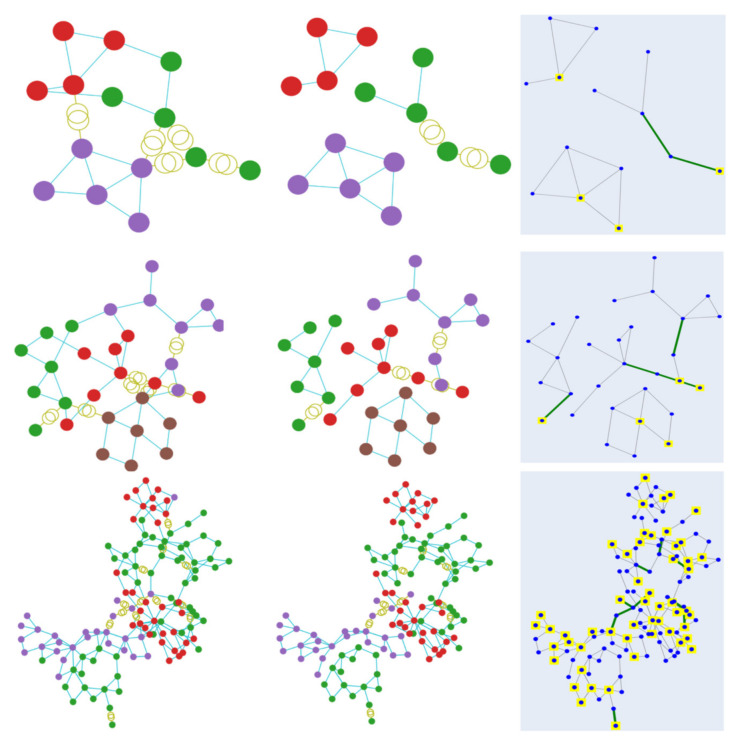
Clustering visualisation of the results obtained by using the GAP loss function. Each color represents a specific cluster. The first row displays the results from case 14, followed by case 30 and case 118.

**Figure 6:**
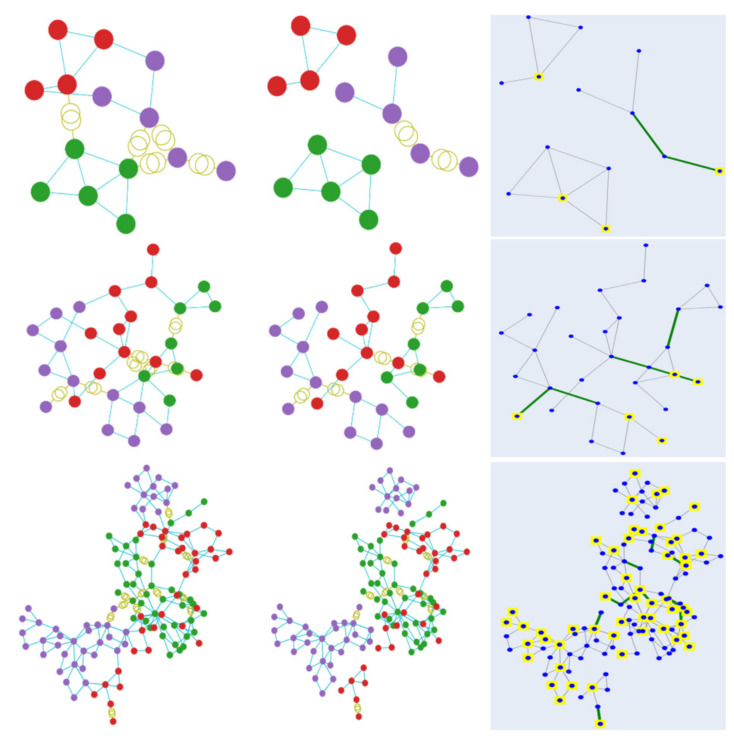
Clustering visualisation of the results obtained by using the min-cut loss function. Each color represents a specific cluster. The first row displays the results from case 14, followed by case 30 and case 118.

Figure 5 illustrates the clustering results obtained by using the GAP loss function. Starting from the top, the depicted outcome is derived from case 14, followed by case 30 and finally case 118. The first column displays the initial clustering result, while the final outcome after merging and cutting is shown in the second column. The images on the third column depict the final results with colored indications to showcase the generators. The clustering results corresponding to the min-cut loss function are displayed similarly in Figure 6. As observed in the figures, both methods result in effective clusterisation in most cases, with only some of the large grid cases resulting in a few elements that are not connected to the rest of the buses in a cluster. In addition, as demonstrated in the third column of each figure, the requirement that every cluster should have at least one generator is sufficiently satisfied by utilising the included generator loss function.

Table 2 provides the numerical results of the proposed methods for test cases 9, 30, ieee30, 57, 118 and 200. These cases represent various scenarios from small to large power grids. The results include the number of lines to be disconnected, the total load-generation imbalance after islanding and the number of islands. As observed in the table, both methods result in the same power imbalance, but the min-cut loss function leads to the fewer number of lines that should be disconnected in order to finish the islanding, resulting in a more efficient process. The results show that there is a cost of applying intentional islanding in the power systems in terms of imbalance between the loads and the available generation, but the methods manage to keep it low, avoiding the creation of highly unstable partitions. As a result, it ensures that the solution will alleviate the impact of the triggering event, without resulting in significant losses of power, especially given the fact that the outcome is obtained very quickly, requiring less than a second, and is dynamic since it is based on real-time system conditions.

Table 3 and Table 4 compare the results of the proposed method with similar approaches found in the literature, on test cases 118 and 200, respectively. In all cases the system is partitioned into four islands at a cost of a load-generation mismatch. The introduced method when using the min-cut loss function, demonstrates a significantly lower imbalance than [10], at the cost of higher number of lines that should be disconnected. The method in [12] provides the result requiring the same number of line disconnections, but is still not able to achieve the imbalance that the proposed technique provides, which ultimately creates the more stable islands.

### 4.3. Ablation Study

**Figure 7:**
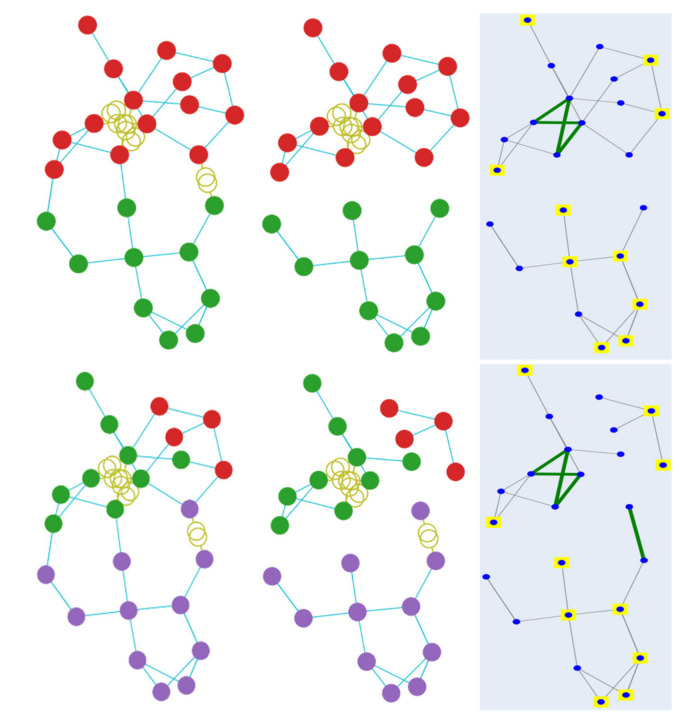
Min-cut clustering results without (**top**) and with (**bottom**) balance cut and bus injection loss functions.

Besides providing the numerical results of the predictions, the results with respect to min-cut loss terms, ignoring the injection balance loss, are also examined. In the conducted experiments, the minimisation of the load-generation imbalance is achieved by the proposed loss function. Figure 7 visualises the clustering result using the min-cut loss on test case 24, in a case with balance cut and bus injection loss and in one without. The images on top provide the results without balance cut and bus injection, while below are the results with all loss functions. It is observed that when using only the min-cut loss function, the result tends to provide a binary result. In contrast, when adding the balance cut, the desired result with the required number of clusters is obtained, making it essential to include the balance cut and bus injection loss when using the min-cut function.

## 5. Conclusions

This paper proposed a novel method that combines the area of graph neural networks with the problem of intentional islanding in power systems. Two deep learning loss functions for graph partitioning were examined and several loss functions were utilised to minimise the load-generation imbalance and improve the final clustering result. The introduced method ensures that each cluster has at least one generator in order to avoid situations where there is no power supply after the cut has been applied and therefore increases the stability of the power system. Experiments demonstrated the effectiveness of the proposed solution and the increased time efficiency, obtaining the outcome in real-time. The results demonstrate that there is a cost of applying the intentional islanding solution when it comes to the imbalance between the load and the available generation, but it is relatively low, avoiding the creation of unstable islands and ensuring that the solution will relieve the effect of the triggering incident, without resulting in significant loss of power. As a result, this implementation can be incorporated in modern electric power and energy systems and enable a rapid-response mechanism for intentional islanding in case of disturbances. The islanding method can be executed on time and calculate a good solution quickly, thus preventing further outages by creating small stable islands with minimum imbalance.

## Figures and Tables

**Table 1:** The output dimensions of the introduced implementation.

Layer	Output Dimension
Graph CNN layer	96
	128
	256
Linear layer	256
	128
	g

**Table 2:** Numerical results of the proposed methods for test cases 9, 30, ieee30, 57, 118, and 200.

Case	Imbalance (MW)		Lines Disconnected	
	GAP	Min-Cut	GAP	Min-Cut
9	4.95	4.95	2	2
30	2.44	2.44	9	9
ieee30	17.56	17.56	9	7
57	27.86	27.86	16	13
118	132.91	132.91	28	21
200	23.42	23.42	33	24

**Table 3:** Comparison of the proposed method with the literature on case 118.

Method	Imbalance (MW)	Lines Disconnected	No. of Islands
Kyriacou et al. [10]	240.03	n/a	4
Proposed (Min-cut)	132.91	24	4

**Table 4:** Comparison of the proposed method with the literature on case 200.

Method	Imbalance (MW)	Lines Disconnected	No. of Islands
Kyriacou et al. [10]	269.01	20	4
Basumallik et al. [12]	35.85	28	4
Proposed (Min-cut)	23.42	28	4

## Data Availability

Not applicable.

## Abbreviations

CNN	Convolutional neural network
GAP	Generalisable Approximate Partitioning
GNN	Graph neural network
MILP	Mixed-integer linear programming
ML	Machine learning
